# Antiphospholipid antibody-related hepatic vasculitis in a juvenile after non-severe COVID-19: a case report and literature review

**DOI:** 10.3389/fimmu.2024.1354349

**Published:** 2024-04-19

**Authors:** Qingyu Li, Jingya Li, Menglan Zhou, Ying Ge, Zhengyin Liu, Taisheng Li, Li Zhang

**Affiliations:** ^1^ Tsinghua Medicine, School of Medicine, Tsinghua University, Beijing, China; ^2^ Peking Union Medical College Hospital, Chinese Academy of Medical Sciences, Peking Union Medical College, Beijing, China; ^3^ State Key Laboratory of Complex Severe and Rare Diseases, Peking Union Medical College Hospital, Beijing, China; ^4^ Department of Infectious Disease, Peking Union Medical College Hospital, Chinese Academy of Medical Sciences, Peking Union Medical College, Beijing, China

**Keywords:** antiphospholipid antibodies, COVID-19, pediatrics, vasculitis, non-thrombotic manifestation, vasculopathy

## Abstract

Antiphospholipid antibodies (aPL) are both laboratory evidence and causative factors for a broad spectrum of clinical manifestations of antiphospholipid syndrome (APS), with thrombotic and obstetric events being the most prevalent. Despite the aPL-triggered vasculopathy nature of APS, vasculitic-like manifestations rarely exist in APS and mainly appear associated with other concurrent connective tissue diseases like systemic lupus erythematous. Several studies have characterized pulmonary capillaritis related to pathogenic aPL, suggesting vasculitis as a potential associated non-thrombotic manifestation. Here, we describe a 15-year-old girl who develops hepatic infarction in the presence of highly positive aPL, temporally related to prior non-severe COVID-19 infection. aPL-related hepatic vasculitis, which has not been reported before, contributes to liver ischemic necrosis. Immunosuppression therapy brings about favorable outcomes. Our case together with retrieved literature provides supportive evidence for aPL-related vasculitis, extending the spectrum of vascular changes raised by pathogenic aPL. Differentiation between thrombotic and vasculitic forms of vascular lesions is essential for appropriate therapeutic decision to include additional immunosuppression therapy. We also perform a systematic review to characterize the prevalence and clinical features of new-onset APS and APS relapses after COVID-19 for the first time, indicating the pathogenicity of aPL in a subset of COVID-19 patients.

## 1 Introduction

Antiphospholipid syndrome (APS) is a systemic autoimmune disorder characteristic of arterial, venous, or microvascular thrombosis, obstetric morbidity, and well-defined non-thrombotic manifestations in the setting of persistent antiphospholipid antibodies (aPL) (1, 2). aPL, composed of a diverse family of acquired autoantibodies, are recognized as causative factors for clinical manifestations of APS (3). Both genetic and environmental elements could exert as precipitating factors for aPL production, with infection being the most prevalent trigger (4–6). In the recent COVID-19 pandemic, the observed high prevalence of aPL has been reported, yet the potential pathogenicity of these antibodies remains uncertain and controversial (7). The molecular mimicry between severe acute respiratory syndrome coronavirus 2 (SARS-CoV-2) viral proteins and native tissues and the neoepitope caused by SARS-CoV-2-induced oxidative stress probably contribute to aPL generation (8). In addition to widely reported aPL-related thrombosis, associated non-thrombotic manifestations are emerging with considerable evidence (9). Compared with adult patients, aPL-related non-thrombotic complications, both criteria and non-criteria, are more frequently presented in pediatric patients (10). aPL-related vasculitis is characterized as the inflammation of vessel walls and is only well-confirmed in pulmonary capillaries as diffuse alveolar hemorrhage (DAH) (11). This rare manifestation can result in occlusion of vessel lumen in the absence of thrombus, which might make it clinically indistinguishable to thrombotic events. Differentiation between thrombotic and vasculitic causes of aPL-related vascular damage is essential for proper therapeutic decision to adequately include immunosuppression therapy (12). Here, we describe an uncommon case of a young girl with aPL-related vasculitis-induced liver infarction after non-severe COVID-19 infection, providing valuable information for development of pathogenic aPL in infectious diseases and aPL-related vasculitic manifestations.

## 2 Case report

**Figure 1:**
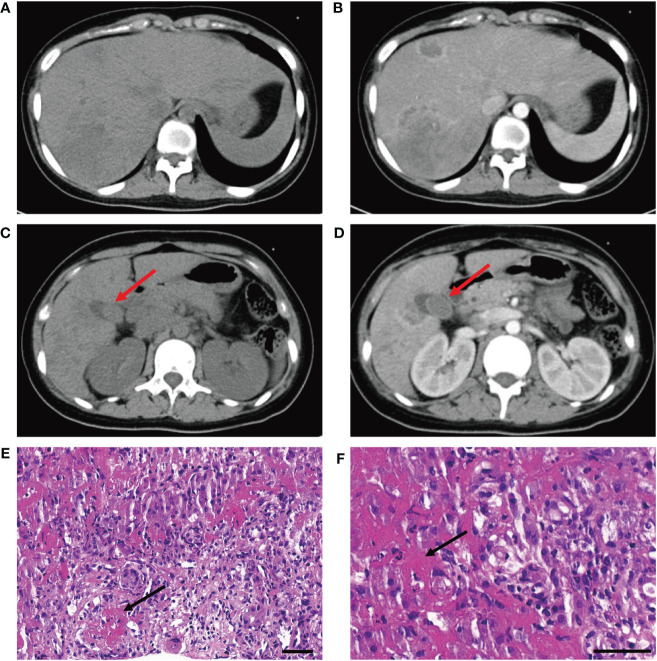
**(A, B)** CT scan without and with contrast demonstrates liver "abscess-like" lesions. **(C, D)** CT scan without and with contrast demonstrates possible cholecystitis (red arrowhead). **(E, F)** Histopathological examination reveals vasculitis of hepatic arteries and resultant liver infarction (black arrowhead) (bar is 50 μm).

**Figure 2:**
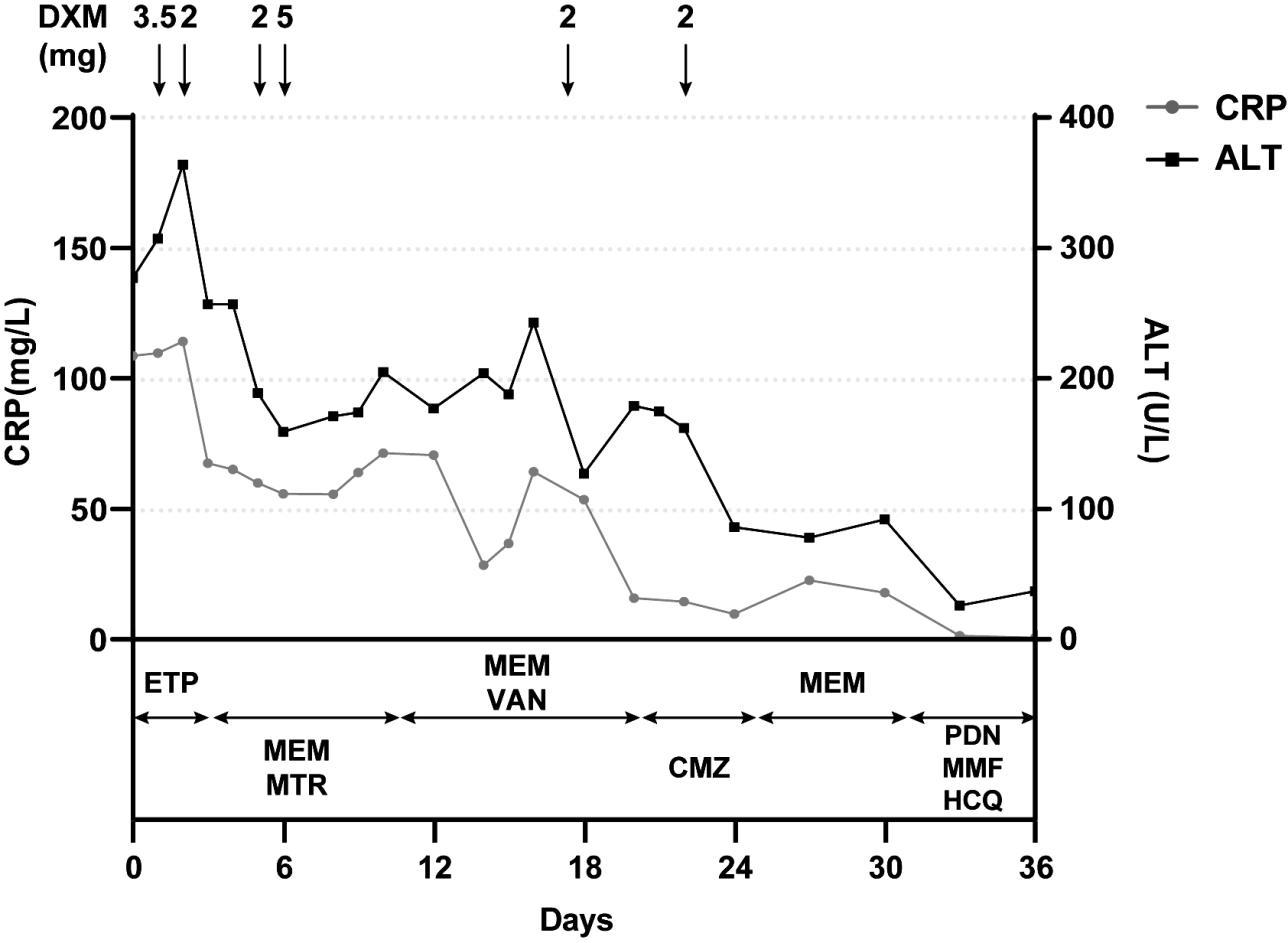
Positive correlation between clinical course and the administration of dexamethasone but not antibiotics. Laboratory reference range for indicators: CRP<3.0 mg/L; ALT: 9–50 U/L. CRP, C-reactive protein; ALT, alanine aminotransferase; DXM, dextromethorphan; ETP, ertapenem; MEM, meropenem; MTR, metronidazole; VAN, vancomycin; CMZ, cefmetazole; PDN, prednisone; MMF, mycophenolate mofetil; HCQ, hydroxychloroquine.

A 15-year-old girl presented to the emergency department with hyperpyrexia and abdominal pain persisting for over a month. Initial laboratory results revealed elevated inflammation markers, liver dysfunction, and prolonged activated partial thromboplastin time (aPTT) (62.2 s; normal: 25–37 s) and prothrombin time (PT) (17.8 s; normal: 11–14 s). There were no bleeding signs clinically. Abdominal CT demonstrated liver “abscess-like” lesions (**Figures 1A, B**) as well as possible cholecystitis (**Figures 1C, D**), and the histopathological examination of liver biopsy specimens confirmed the acute hepatic necrosis. An empiric anti-infective therapy was initiated with intravenous ertapenem and changed to meropenem and metronidazole later. Vitamin K and plasma transfusion were applied for correction of coagulation disorders but turned out to be ineffective. Possible pathogens were under intense exploration, but all proved negative after following tests: traditional microbiologic culture and metagenomic next-generation sequencing of peripheral blood samples and liver biopsy specimens; serology screenings for fungi, SASR-CoV-2, hepatitis viruses, TORCH pathogens, Leishmania, and mycobacteria tuberculosis; Epstein–Barr virus DNA analysis; and microscopic examination of parasites in stool samples. In addition, tumor marker analysis, bone marrow examination, specific staining of liver biopsy specimens, and ceruloplasmin test showed no abnormalities. The efficacy of anti-infective therapy was undetermined with fluctuating inflammation markers and unrelieved abdominal pain. Elevated D-dimer (14.87 mg/L; normal: 0–0.55 mg/L), fibrin and fibrinogen degradation products (FDP) (27.6 μg/mL; normal: 0–5 μg/mL), and fibrinogen (7.52 g/mL; normal: 1.8–3.5 g/mL) were also indicated. Repeated CT demonstrated enlargement or reduction of some liver lesions, as well as the emergence of new lesions. The antibiotics were improved to intravenous ertapenem and vancomycin after the re-elevation of C-reactive protein. Unexpectedly, her symptoms worsened with a re-elevated fever peak and persistent coagulation disorders (**Figure 2**).

The young girl had a medical history of mild SARS-CoV-2 Omicron variant infection, presented with only nasal congestion and fatigue. The onset of abdominal pain occurred 7 days after testing negative for SARS-CoV-2 antigen and complete remission of COVID-19 symptoms. As the efficacy of low-dose dexamethasone given during plasma transfusion could not be excluded for transient improvement of the patient (**Figure 2**), an immune dysregulation secondary to infection was considered. Serologic testing of antinuclear antibodies and autoimmune hepatitis antibodies only revealed the presence of low titers of antinuclear antibody (1:80) and smooth muscle antibody (1:80). Autoimmune liver diseases were ruled out. Antibodies associated with other systemic autoimmune diseases were comprehensively evaluated, and the presence of IgG anticardiolipin (aCL) and IgG anti-β2-glycoprotein I (anti-β2GPI) antibodies, as well as lupus anticoagulant (LA), was demonstrated (**Table 1**). Other autoantibodies were undetected. The aPTT and PT correction tests showed negative results. Consecutive examinations demonstrated disease progression following changes of aPL titers, and hepatic lesions were considered to be related to aPL. Considering the hypercoagulable state of pathogenic aPL, the patient underwent CT scan, MRI, MRA, and vascular ultrasound and multiple small acute to subacute cerebral infarcts were indicated. No thrombus and other abnormalities were detected. The liver biopsy specimen was histologically re-evaluated. Immune infiltration with a large number of neutrophils and fibrinoid necrosis were evident in some small- and medium-sized arteries, and adjacent hepatic tissues underwent ischemic infarction consequently (**Figures 1D–F**). The patient traversed the most severe phase because of administered low-dose dexamethasone. Further therapy included 40 mg prednisone once a day, 500 mg mycophenolate mofetil (MMF) twice a day, and 200 mg hydroxychloroquine twice a day for immunosuppression, along with 100 mg aspirin once a day to prevent future thrombosis. The patient were discharged from the hospital with reduced inflammation markers, diminished abdominal pain, and healed liver lesions as shown in CT examination. The administration of MMF, hydroxychloroquine and aspirin remained unchanged after discharge, whereas prednisone was gradually tapered. The dose reduction proceeded at 5 mg per week until reaching a daily dose of 20 mg, followed by a weekly reduction of 2.5 mg until reaching a daily dose of 15 mg. Scheduled follow-up appointments were conducted. Six months after discharge, the patient discontinued medication autonomously and subsequently experienced a relapsed right-upper quadrant pain with re-elevated aPL titers and significantly prolonged aPTT (**Table 1**). D-dimer, FDP, and fibrinogen were within normal ranges. Resumption of treatment yielded amelioration. Considering the persistence of medium-to-highly positive aCL and LA for over 12 weeks, as well as aPL-related hepatic vasculitis and cerebral infarction, the diagnosis was made as highly probable APS with vasculitis as a non-criteria manifestation.

**Table 1:** Disease parameters and titers of autoantibodies.

Days post hospital admission	Day 11	Day 22	Day 30 (discharge)	Day 76 (follow-up)	Day 181 (follow-up)	Day 215 (relapse)	Reference range
aPTT (s)	45.2	71.9	53.8	/	/	50.6	23.3–32.5
PT (s)	20.1	15.1	13.3	/	/	14.4	10.4–12.6
INR	1.70	1.25	1.09	/	/	1.24	0.86–1.14
ANA	(+) S1:80	(+) S1:80	/	(-)	(-)	(-)	(-)
IgG anti-β2GPI* (U/mL)	(+) 34.3	(+) 42.8	(+) 33.6	(-)	(-) 11.6	(+) 29.1	<20
IgG aCL* (U/mL)	(+) 39.8	(+) 51.0	(+) 41.3	(+) 12.4	(+) 12.3	(+) 33.2	<10
LA†	2.36	/	2.85	/	1.64	2.09	(-)

(/), not tested.

aPTT, activated partial thromboplastin time; PT, prothrombin time; INR, international normalized ratio; ANA, anti‐nuclear antibody; anti-β2GP I, anti‐β2‐glycoprotein I; aCL, anticardiolipin; LA, lupus anticoagulant.

*aCL and anti-β2GPI were measured using chemiluminescent immunoassay (CLIA) (iFlash CLIA kits provided by YHLO Biotech Co., Shenzhen, China). According to the manufacturer’s instructions, a titer exceeding 10 U/mL was defined as medium or high level for aCL, while a titer exceeding 20 U/mL was defined as medium or high level for anti-β2GPI. The assay demonstrated robust sensitivity and specificity in previous cohort study (13).

†LA assay was performed based on a three-step procedure with two screening test systems recommended by the International Society of Thrombosis and Hemostasis guidelines (14).

## 3 Discussion

Compared with the updated Sapporo criteria with only vascular thrombosis and pregnancy morbidity as diagnostic manifestations of APS (1), the new 2023 ACR/EULAR criteria has introduced several well-defined non-thrombotic manifestations into the clinical criteria for APS classification, including microvascular diseases, cardiac valve diseases, and thrombocytopenia (2). A progression and advancement of the comprehension of aPL-related clinical manifestations is indicated. However, limitations still exist as patients with criteria aPL and comparatively uncommon non-thrombotic manifestations and patients with fulfillment of clinical criteria but seronegative conventional aPL might be inadequately excluded. These conditions are therefore suggested to be referred as “probable APS” or “non-criteria APS” (15). Our case met the laboratory criteria based on persistence of medium-to-highly positive aCL and LA. The complete and sustained remission of hepatic vasculitis was achieved only when aPL were managed at lower titers with pathogenic effects effectively suppressed. The development of cerebral infarction happened in the setting of highly positive aPL and in the absence of other vascular risk factors. Hepatic vasculitis and cerebral infarction were therefore considered to be associated manifestations. The pathophysiology of cerebral infarcts was undetermined, yet the remarkably elevated D-dimer and FDP suggested a possibility of thrombotic events. Accordingly, our case was assessed as highly probable APS with aPL-related hepatic vasculitis as a non-criteria manifestation, and the development of pathogenic aPL was associated with prior COVID-19 infection.

Infections have been implicated in induction of autoimmunity including aPL production (16), with the recent COVID-19 pandemic being no exception (7). A large number of studies have reported high prevalence of aPL (5%–71%), both criteria and non-criteria types, in COVID-19 patients (7, 17). Several potential mechanisms have been proposed but require further validation (8). Molecular mimicry supposes that the S1 and S2 subunits of the SARS-CoV-2 viral S protein might form a phospholipid-like epitope shared with native tissues, triggering aPL production and provoking an immunogenic response (18–20). The neoepitope model posits that oxidative stress induced by SARS-CoV-2 can alter the conformation of β2GPI (21, 22) and create a neoepitope for antibody generation (23).

Despite the observed high prevalence, the pathogenicity of COVID-19-associated aPL remains uncertain and controversial. To explore the potential roles of aPL, numerous studies have analyzed the correlations of aPL and clinical manifestations in COVID-19 patients, yet a consensus could not be reached. COVID-19-associated aPL were demonstrated to be natural or nonpathogenic in most studies (24–50), which was also shown in the largest meta-analysis published in 2021 (51). Additionally, anti-β2GPI in COVID-19 was reported to rarely (5%) recognize domain I of β2GPI, the molecular region most commonly associated with pathogenicity (24). On the contrary, associations of aPL with disease severity and thrombosis in COVID-19 patients were also reported (52–67). Notably, the largest cohort study demonstrated a correlation between the presence of aCL or IgA anti-β2GPI and thrombotic events (65). IgG antibodies purified from COVID-19 patients with high aPL titers were found to trigger neutrophil extracellular trap release and potentiate thrombosis in mice, similarly to IgG isolated from individuals with definite APS (59). Additionally, infections have been reported as the most common causative factor of catastrophic APS (CAPS), suggesting that infection-induced aPL could exhibit biological activity in a subset of patients (6). Several theories have been proposed to explain the heterogeneity. The “two hits” theory holds that aPL (first hit) induce a thrombophilic state, but clotting requires additional thrombophilic condition (second hit), often involving an innate immunity activator like inflammation, infection, or surgery (3). Furthermore, infections are proposed to more likely trigger APS in individuals with genetic propensity, immune defects, or hormonal abnormalities (16). Therefore, the pathogenicity of aPL exhibits heterogeneity across COVID-19 patients and susceptible individuals with predisposing factors might present aPL-related manifestations in the presence of COVID-19-associated aPL.

Albeit the intense exploration of aPL in COVID-19 patients by multiple studies, most of them neither specify the duration of aPL positivity nor subgroup patients according to antibody levels. All COVID-19 patients with positive aPL were incorporated, and individuals with or without pathogenic aPL were merged together for characterization and analysis, contributing to the debatable pathogenicity of aPL. Systematic analyses based on these studies could not reveal the prevalence and features of patients developing pathogenic aPL after COVID-19. Conversely, new-onset APS in COVID-19 patients have also been reported, wherein persistently high-titer aPL, associated thrombotic and non-thrombotic manifestations, and recovery following treatments based on APS management guidelines substantially indicate the pathogenicity of aPL. Therefore, new-onset APS cases could be a narrow representative of COVID-19 patients with pathogenic aPL. We systematically reviewed the literature of relevant cases up to February 2024 using PubMed and EMBASE to analyze the APS onset after COVID-19 for the first time. The cases reported as APS after COVID-19 infection with sufficient information to meet the updated Sapporo criteria or the new 2023 ACR/EULAR criteria (1, 2) or with limited information but compelling evidence to support the diagnosis of APS were included. The former and the latter were annotated as definite APS and highly probable APS respectively. Nine cases (68–76) were identified and evaluated together with our case (**Table 2**). The patients ranged from 15 to 89 (mean = 41.10) years of age and most of the patients were female, consistent with the epidemiology of APS that is more common in middle-aged women (82). According to WHO-issued guidelines (83), COVID-19 severity of these cases encompassed a spectrum from non-severe to critical, indicating that aPL-related manifestations did not merely develop on the basis of cytokine storms in critical patients. The time interval from COVID-19 to the onset of APS varied from 7 to 41 days (mean = 18 days) and a probably more frequent occurrence during the convalescent period was suggested. Definite or probable CAPS was reported in three cases (30%), significantly higher than the approximate 1% incidence of CAPS in all APS patients (84). Thrombosis and corresponding organ infarctions (80%) were the most common manifestation, followed by thrombocytopenia (30%). There were 12 patients mentioned in four cohort studies who also fulfilled the inclusion criteria but were not included due to the lack of individualized information (50, 64, 67, 77–81, 85). In addition to newly diagnosed APS, five cases have reported relapses of completely remitted APS following COVID-19 infections (**Table 2**), reiterating SARS-CoV-2 as a potential trigger for pathogenic effects of aPL and exacerbation of APS in some patients. Here, we report the first case of developing pathogenic aPL in a juvenile after non-severe COVID-19, diagnosed as highly probable APS. Notably, the absence of any medical history in the patient alerts the possibility of developing severe aPL-related symptoms following non-severe COVID-19 infection in previously healthy individuals, which was also indicated in a healthy woman developing obstetric APS (OAPS) after non-severe COVID-19 infection (73).

**Table 2:** Demographic and clinical characteristics of APS after COVID-19.

Patients	Age (years)	Sex	Medical history of autoimmune diseases	COVID-19 severity	Time from COVID-19 to onset of APS symptoms (days)	Antiphospholipid antibodies	Primary manifestations	Inclusion criteria
Definite new-onset APS								
1 (68)	89	Female	None	Severe	14	IgM aCL IgM anti-β2GP I LA	Deep venous thrombosis	Thrombosis Persistent aPL>12 weeks
2 (69)	46	Female	AIH	Severe	41	aCL LA	Adrenal infarcts	Thrombosis Persistent aPL>12 weeks
3 (70)	22	Female	SLE	Non-severe	7	IgG aCL IgG anti-β2GP I LA	Deep vein thrombosis Thrombocytopenia Pulmonary emboli	Thrombosis Thrombocytopenia Persistent aPL >12 weeks
4 (71)	63	Female	SLE	Severe	14	aCL LA	Thrombosis Refractory thrombocytopenia	Thrombosis Thrombocytopenia Persistent aPL >12 weeks
5 (72)	36	Female	SLE	N/A	N/A	aCL anti-β2GP I LA	Budd–Chiari syndrome Thrombocytopenia	Budd–Chiari syndrome Thrombocytopenia Persistent aPL>12 weeks
6 (73)	39	Female	None	Non-severe	N/A	IgG aCL IgG β2GP I	Preeclampsia HELLP syndrome	Preeclampsia Persistent aPL>12 weeks
Highly probable new-onset APS								
7 (74)	47	Female	None	Non-severe	30	IgG aCL, IgM aCL IgG anti-β2GP I, IgM anti-β2GP I	Splenic infarcts Bilateral renal infarcts	Thrombosis Double positive aPL with remarkably high titers
8 (75)	26	Male	None	Critical	7	aCL anti-β2GP I LA	Splenic infarcts Celiac trunk and superior Mesenteric artery thrombosis	Thrombosis Triple positive aPL with high titers
9 (76)	28	Female	SLE	Severe	N/A	IgM aCL IgM anti-β2GP I	Diffuse alveolar hemorrhage	Diffuse alveolar hemorrhage Double positive aPL with high titers
10 (our case)	15	Female	None	Non-severe	13	IgG aCL IgG anti-β2GP I LA	Liver infarcts Cerebral infarcts	aPL-related non-criteria manifestation Probable thrombosis Persistent aPL >12 weeks
Relapse of APS								
11 (77)	51	Female	Remitted APS	Severe	N/A	N/A	Severe thrombocytopenia Suspicious pulmonary hemorrhage	Onset of APS manifestations after COVID-19
12 (78)	38	Female	Remitted APS	Severe	12	IgG aCL, IgM aCL IgG anti-β2GP I, IgM anti-β2GP I	Adrenal glands hemorrhage Limb arterial ischemia	Onset of APS manifestations after COVID-19
13 (79)	64	Female	Remitted APS	Critical	N/A	IgG aCL, IgA aCL IgG anti-β2GP I, IgA anti-β2GP I LA	Stroke Venous thrombosis Adrenal hemorrhage	Onset of APS manifestations after COVID-19
14 (80)	66	Female	Remitted APS	Severe	5	IgG aCL, IgM aCL IgG anti-β2GP I LA	Venous thrombosis Adrenal hemorrhage	Onset of APS manifestations after COVID-19
15 (81)	43	Female	Remitted APS	Non-severe	14	N/A	Stroke	Onset of APS manifestations after COVID-19

N/A, not available.

Remitted APS: years of remission under effective treatment.

APS, antiphospholipid syndrome AIH, autoimmune hepatitis; SLE, systemic lupus erythematosus; anti-β2GP I, anti‐β2‐glycoprotein I; aCL, anticardiolipin; LA, lupus anticoagulant HELLP: hemolysis, elevated liver enzymes and low platelet; aPL, antiphospholipid antibodies.

Differences in distribution, clinical presentations, and outcomes exist between pediatric and adult APS (86). Compared with adult patients, juvenile patients more frequently exhibit non-thrombotic aPL-related manifestations (10). A study including 121 juveniles fulfilling the updated Sapporo criteria demonstrated a high prevalence of associated non-thrombotic manifestations with neurologic, hematologic, and skin disorders being the most common (87). Non-thrombotic manifestations sometimes precede later thrombotic events (88), leaving pediatric patients with isolated non-thrombotic manifestations being inadequately excluded from APS patient population. Accordingly, diagnostic criteria for definite APS are inapplicable in juveniles. Recommendations for management of pediatric APS published by SHARE initiative advocated for the incorporation of non-criteria manifestations into classification criteria for pediatric APS (86). Therefore, recent studies in pediatric APS have concentrated mainly on pathogenic aPL and associated manifestations rather than definite APS. A study of pediatric APS including definite and probable cases revealed high percentage of hematologic and skin disorders (89). Moreover, another analysis of children with medium or highly positive aPL suggested that more than half exhibited non-thrombotic aPL-related manifestations alone (90).

In our case, the histopathology of liver biopsy specimens revealed immune infiltration and fibrinoid necrosis of arteries without granulomatosis, indicating the existence of hepatic vasculitis that has not been reported in association with pathogenic aPL before. The resultant occlusion of arteries gave rise to liver ischemic necrosis in the absence of any notable thrombus or microthrombus. The patient was successfully treated with immunosuppression, further supporting a vasculitic other than thrombotic etiology.

Although debatable, vascular lesions raised by aPL could be inflammatory. DAH, characterized by bleeding into the alveolar space resulting from disruption and injury of pulmonary microcirculation, represents a genuine inflammatory complication of APS and has been included into clinical criteria for APS in the 2023 ACR/EULAR criteria (2, 91). Several studies have investigated the primary APS-associated DAH in recent years (11, 92–94). Surgical or transbronchial biopsies were performed in 20 cases and capillaritis without thrombus or microthrombus was histologically documented in 11 of them (55%), indicating an isolated inflammatory vasculopathy in DAH development. The recommended and efficient treatment of DAH in APS with glucocorticoids and immunomodulatory agents re-emphasizes an inflammatory instead of thrombotic etiopathology of DAH (91). Additionally, mesenteric vasculitis is considered to be one of aPL-related microvascular manifestations as well (95). Sporadic cases with authentic associated vasculitic manifestations have also been reported in cerebral (96), renal (97), aortic (98), and cutaneous (99) vasculature, and no local thrombus or microthrombus was noted in these inflammatory lesions.

Therapy for APS is diverse and individualized based on a broad spectrum of manifestations. Long-term oral anticoagulants like warfarin are recommended for thrombotic APS (100), and alternative therapies such as extended therapeutic dose of low-molecular-weight heparin can be utilized for patients with recurrent thrombotic events despite warfarin (101). For aPL carriers with high-risk profiles or OAPS patients, low-dose aspirin is proposed for primary thrombosis prevention, particularly in individuals with additional vascular risk factors (100, 102). Glucocorticoids; immunomodulatory agents including MMF, cyclophosphamide, and azathioprine; and B-cell-modulating agents like rituximab and belimumab, are recommended in cases with non-thrombotic manifestations (103, 104). Notably, these recommendations, based on adult-derived studies, might be improper for pediatric populations due to differences in physiological conditions, metabolic capacities and duration of medication. Additionally, the low prevalence and heterogeneity of APS in juveniles impede the formation and limit the strength of evidence-based guidelines (86, 105), contributing to substantial variations in treatment regimens that are mostly based on physicians’ experience or observational studies.

In our case, aspirin was administered without anticoagulants. The decision was made based on vasculitis-induced hepatic infarction as the major clinical presentation, repair of cerebral lesions with indefinite pathology before systemic treatment, impaired liver synthetic function for coagulation factors, and the absence of other thrombosis risk factors. As concurrent thrombosis risk factors like arterial hypertension, hyperlipidemia, atherosclerosis and smoking are rarely observed in younger subjects, long-term anticoagulation therapy is not indicated in pediatric thrombotic APS patients harboring discontinuous aPL (106–108). Likewise, immunosuppressive therapy in our case reduced aPL titers close to baseline levels and suppressed their pathogenicity, reminiscent of patients with discontinuous aPL. Combined together, anticoagulants were not administered temporarily. However, the patient underwent intensive and regular follow-up to monitor for emergence of any additional thrombosis risk factors, in which scenario, anticoagulants would be introduced as a replacement of aspirin.

aPL-related thrombosis and vasculitis can cause similar clinical presentations including organ infarctions, whereas the treatment decision is different due to the underlying pathologies. The histopathologic results helped us to confirm the inflammatory vasculopathy and guided the treatment to adequately include immunosuppression comprising glucocorticoids and immunomodulatory agents. Therefore, when no thrombus is detected by non-invasive examinations, biopsy for confirmation of the underlying vasculopathy is suggested in APS, if possible and especially when liver is involved.

Our treatments were individualized based on an atypical case. Although the outcome was favorable, the efficacy and safety of aspirin without anticoagulants require further validation during extended follow-up. We merely recommend the addition of immunosuppressants to conventional therapy for managing aPL-related vasculitis.

## 4 Conclusion

Given the perplexing and contentious nature of aPL produced during infections, the COVID-19 pandemic provides a distinctive opportunity to comprehensively assess this issue. The literature review and analysis evaluate the onset and relapse of APS after COVID-19 infection, suggesting that SARS-CoV-2-triggered aPL may exert pathogenic effects in a subset of COVID-19 patients.

Altogether, we endorse the hypothesis that pathogenic aPL can raise vascular damage manifested as vasculitis other than thrombosis, conveying distinct therapeutic considerations to include immunosuppression therapy. In addition to vasculitis, other forms of vascular lesions including proliferative vascular diseases have also been described in APS (109), extending the spectrum of vascular changes associated with pathogenic aPL. Such cumulative evidence supports the statement that the nature of APS should be extended to both thrombophilia and vasculopathy.

## Data availability statement

The original contributions presented in the study are included in the article/supplementary material. Further inquiries can be directed to the corresponding author.

## Ethics statement

Written informed consent was obtained from the individual(s) for the publication of any potentially identifiable images or data included in this article.

## Author contributions

QL: Conceptualization, Formal analysis, Investigation, Writing – original draft. JL: Conceptualization, Formal analysis, Investigation, Writing – original draft. MZ: Writing – review & editing. YG: Writing – review & editing. ZL: Funding acquisition, Writing – review & editing. TL: Funding acquisition, Writing – review & editing. LZ: Resources, Supervision, Writing – review & editing.
